# The Ca^2+^ Influence on Calmodulin Unfolding Pathway: A Steered Molecular Dynamics Simulation Study

**DOI:** 10.1371/journal.pone.0049013

**Published:** 2012-11-07

**Authors:** Yong Zhang, Jizhong Lou

**Affiliations:** Laboratory of Non-Coding RNAs, Institute of Biophysics, Chinese Academy of Sciences, Beijing, China; University of Oldenburg, Germany

## Abstract

The force-induced unfolding of calmodulin (CaM) was investigated at atomistic details with steered molecular dynamics. The two isolated CaM domains as well as the full-length CaM were simulated in N-C-terminal pulling scheme, and the isolated N-lobe of CaM was studied specially in two other pulling schemes to test the effect of pulling direction and compare with relevant experiments. Both Ca^2+^-loaded CaM and Ca^2+^-free CaM were considered in order to define the Ca^2+^ influence to the CaM unfolding. The results reveal that the Ca^2+^ significantly affects the stability and unfolding behaviors of both the isolated CaM domains and the full-length CaM. In Ca^2+^-loaded CaM, N-terminal domain unfolds in priori to the C-terminal domain. But in Ca^2+^-free CaM, the unfolding order changes, and C-terminal domain unfolds first. The force-extension curves of CaM unfolding indicate that the major unfolding barrier comes from conquering the interaction of two EF-hand motifs in both N- and C- terminal domains. Our results provide the atomistic-level insights in the force-induced CaM unfolding and explain the observation in recent AFM experiments.

## Introduction

**Figure 1:**
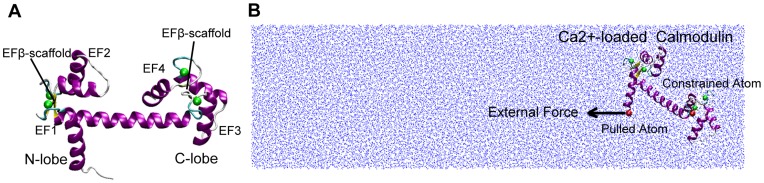
The CaM structures and the simulation system setup. (A) the structure of the Ca2+-loaded full-length CaM, including two domains: N-lobe and C-lobe, and four EF-hand motifs: EF1, EF2, EF3, and EF4. EFβ-scaffold is a short β-sheet coupled two EF-hand motifs. (B) The sketch map of SMD simulation for Ca^2+^-loaded full-length CaM. Two red balls represent the pulled and constrained atoms, respectively. Black arrow represents the direction of the pulling force. The blue dots represent the water solvent. The green balls in both panels represent the Ca^2+^ ions.

The small two-domain protein calmodulin (CaM) is a ubiquitous intracellular Ca^2+^ binding protein, in which each domain contains a pair of coupled EF-hand motifs and binds two Ca^2+^ ions [1], [2], as shown in Fig. 1A. The EF-hand motif, which has the helix-loop-helix design and is the most common Ca^2+^ binding motif, generally undergoes large conformational changes upon Ca^2+^ binding. In the Ca^2+^-loaded CaM, EF-hands adopt an open conformation and CaM presents a relative rigid and stable holo state, while in the Ca^2+^-free form of CaM, EF-hands adopt a closed conformation and CaM presents a flexible apo state [3], [4], [5]. Ca^2+^-loaded CaM can bind to more than 300 target proteins to regulate the downstream biological processes [6], [7], [8].

Due to the importance of CaM in the Ca^2+^ signals pathway, it has been the focus of extensive studies in the past several decades, including many versions of its X-ray or NMR structures [9], [10], [11], [12], its metal-binding properties [4], [13], [14], the interaction with its target proteins [7], [15], and its stability and dynamics in water environment and so on. Of interest, several published papers are related with the folding/unfolding properties of CaM [16], [17], [18], [19], [20], [21]. Bayley’s lab makes use of the chemical (urea) and thermal denaturation method to investigate the stability of holo- and apo-CaM [16], [17], and their results demonstrated that for the Ca^2+^-free CaM, C-domain in intact CaM is less stable than when isolated and N-domain in intact CaM is more stable than when isolated, but for the Ca^2+^-loaded CaM, N-domain in intact CaM is less stable than when isolated, and C-domain in intact CaM is more stable than when isolated. The C-terminal domain has been suggested to be inherently less flexible than the N-terminal domain [22]. The small-angle X-ray scattering method was also used to study the denaturation of CaM induced by urea and the results suggested that there exist the unfolding intermediate which is an asymmetric dumbbell-like structure, one in the folded and one in the unfolded state [18]. C.K. Johnson’s lab used single-pair fluorescence resonance energy transfer (spFRET) measurements to characterize denatured and partially denatured states of both Ca^2+^-loaded and Ca^2+^-free CaM, and they suggested a model for stepwise unfolding of CaM in which the domains of CaM unfolded sequentially [19]. Moreover, the thermal unfolding simulation of apo-CaM using leap dynamics also demonstrated the two domains unfolded sequentially [20]. Coarse-grained molecular dynamics was used to explain the folded/unfolded transition of CaM C-terminal domain [21]. These studies all revealed that Ca^2+^ could affect the folding/unfolding of CaM remarkably, and two domains of CaM would unfold sequentially. However, little is known about the folding/unfolding behaviors of CaM in the atomistic level because the methods mentioned above could not address the detailed structural change during the folding/unfolding process.

On the other hand, in recent years the single-molecular mechanical spectroscopy techniques had become apparent to investigate the unfolding behavior of proteins. Rief and its colleges study the unfolding pathway of CaM using the low-drift atomic force microscope (AFM) method [23]. Ikai investigated the unfolding mechanics of holo- and apo- CaM by AFM method [24]. These studies analyzed the unfolding pathway of CaM based on the force-extension trace which can easily be obtained using AFM method. The AFM method has an inevitable limitation that the atomistic-level view of the process of forced-induced unfolding could not be addressed. Steered molecular dynamics (SMD), which correspond closely to the AFM experimental situation, is a proper method to provide the information on the process at atomistic-level resolution that cannot be obtained from experiment. Many researchers have make use of SMD method to discover the unfolding or unbinding events of protein successfully [25], [26], [27].

In this study, we will use the steered molecular dynamics method to investigate the force-induced unfolding pathway of the isolated domains of CaM and intact CaM in the presence and absence of Ca^2+^. Both N-C-terminal pulling scheme and two non-N-C-terminal pulling geometries are applied to N domains of CaM to verify whether the different pulling geometries converge onto a common pathway. The calculated force-extension curves of Ca^2+^-loaded CaM are in agreement with that obtained experimentally [23], [28]. Our simulation results suggest that the unfolding order of N- and C-terminal domains are related with the Ca^2+^ binding states, and the interactions between the two EF-hand motifs compose the main energy barrier on CaM unfolding.

## Models and Methods

### System Preparation and Equilibration

The initial structures of Ca^2+^-loaded and Ca^2+^-free CaM are obtained form the Protein Data Bank (Ca^2+^-loaded CaM coded by 1CLL [9], Ca^2+^-free CaM coded by 1CFD [10]), which have the same sequences. Based upon the structural information, CaM is divided into three parts: N-lobe (Residues 1–74) which includes EF-hand I (EF1 for short, Residues 11–40) and EF-hand II (EF2-for short, Residues 47–74), the central linker (Residues 75–82) and C-lobe (Residues 83–148) which includes EF-hand III (EF3 for short, Residues 84–113) and EF-hand IV (EF4 for short, Residues 120–148), as shown in Fig. 1A. In order to study the unfolding pathway of CaM systematically, six systems are constructed which are N-lobe, C-lobe and the full-length molecule of Ca^2+^-loaded and Ca^2+^-free CaM, respectively.

After adding of the missing hydrogen atoms, the protein was immersed in a TIP3P water box [29]. Sodium and chlorine ions were included to neutralize the systems (ions concentration: 0.15 M). The simulated full-length Ca^2+^-loaded CaM system is shown in Fig. 1B, and the detailed information of the six systems is listed in Table S1,. Energy minimization of 8000 steps was first performed using conjugate gradient method. The subsequent systems were then equilibrated for 14 ns in NPT ensemble.

During the simulation, Particle Mesh Ewald (PME) method with grid spacing of 1 Å was used for long-range electrostatic interactions [30]. VDW interactions were computed using the switch method, in which the Lennard-Jones (LJ) 6–12 potential is calculated normally within the 10Å, and switches off smoothly between 10 to 12 Å. SHAKE method was used on all hydrogen-containing bonds to allow a 2 fs time step in the equilibration simulations [31]. Temperature (300 K) was controlled using Langevin dynamics method and the damping coefficient was set to 2/ps. Pressure (1 atm) was controlled using the Langevin piston Nosé-Hoover method. Potential energy, temperature and pressure of all systems show a nice stability during the equilibration periods.

### Steered Molecular Dynamics

The equilibrated snapshots at 4 ns and 14 ns for all six systems and two extra snapshots for the Ca^2+^-loaded and Ca^2+^-free full-length CAM were used as the starting conformations of SMD simulations. The N-C-terminal pulling geometries were applied to all six systems, in which a dummy spring was attached to Cα atom of N-terminal residue and moved with a constant velocity to generate the pulling force, while the Cα atom of C-terminal residue is constrained. The external force is directed along the vector from the pulled atom to the constrained atom, as shown in Fig. 1(B). For N-lobe, two more non-N-C-terminal pulling geometries are performed to test the influence of pulling location to N-lobe unfolding and explain the relevant AFM experimental results in details [28], in which the pulling force were applied on residues 17 or 38 respectively whilst keeping the C-terminal residue of N-lobe constrained, as shown in Fig. S1. The force constant (k) of the dummy spring was set to 0.1 kcal/mol*Å^2^ (∼7 pN/Å), corresponding to a thermal fluctuation of the pulling atom of 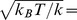
0.03Å, and the moving velocity of the spring was choose to be 5 Å/ns for most cases. Lower pulling speed at 1 Å/ns was also used for the full-length CaM to test the validity of the simulations. In SMD simulations, the step size was set to 1fs with the SHAKE method turned off. The structure snapshots and the external forces were recorded every 1 ps. The extension of the protein is defined as the end-to-end distance between the pulled Cα atoms and the constrained Cα atom.

### Tools and Software

All energy minimizations and molecular dynamics simulations were carried out using the software package NAMD2.7 [32] and CHARMM22 force field with CMAP corrections [33]. System preparations, trajectory analyses and illustrations were performed using the VMD program [34].

## Results

### Equilibration

As reflected by relatively constant backbone RMSD values from their initial structures, all six systems we simulated reached equilibrium quickly during the conventional molecular dynamics equilibration (Fig. S2). The backbone RMSDs of three Ca^2+^-free systems were larger than their corresponding Ca^2+^-loaded systems, indicating that Ca^2+^-free CaM is more flexible than Ca^2+^-loaded one and the presence of Ca^2+^ could stabilize the structure of CaM in solution. Most physical properties, including the backbone hydrogen bond networks of the EFβ-scaffold coupled two EF-hand motifs (denoted as “backbone H-Bond” in the following), the contact areas of two EF-hand motifs, and the gyration radius of both EF-hand motifs, also remained stable (Table S2 and Table S3). The distances between Ca^2+^ cations and their coordinating atoms are ∼2.2–2.6Å during the equilibration period for all three Ca^2+^-loaded systems. The equilibrated snapshots at 4 ns and 14 ns for all systems are used as the initial structures of the SMD simulations. For full-length CaM systems, the snapshots at 3.5 ns and 3.75 ns were also used as the SMD starting point.

### Forced Unfolding of the Isolated N- or C-lobes

The N- and C-lobes of CaM have distinct physical characteristics [35], [36], [37], and experimental studies also showed that the two domains fold and unfold independently [16], [23]. Therefore we firstly simulated the unfolding processes of the isolated domains of CaM.

**Figure 2:**
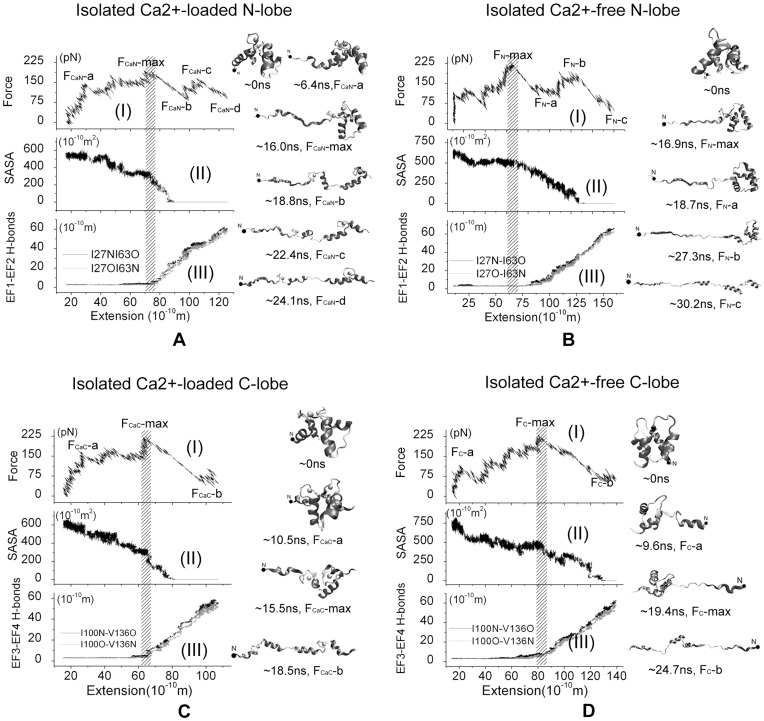
MD simulation results of the isolated CaM domains using 4 ns snapshot of equilibration as the initial structure and ν = 5 Å/ns. A) Ca^2+^-loaded N-lobe, B) Ca^2+^-free N-lobe, C) Ca^2+^-loaded C-lobe, D) Ca^2+^-free C-lobe. On the left, (I): Force-extension curve; (II) Contact area of two EF-hand motifs; (III) Backbone hydrogen bonds of EFβ-scaffold coupled two EF-hand motifs. On the right, the snapshots of the forced-unfolding processes. The external force was applied on the atoms presented with the black solid circle in N-terminal end and another end of protein is constrained.

The force-extension curves of the SMD simulations starting form different conformations show similar features and the results of Ca^2+^-loaded and Ca^2+^-free N- and C-lobe unfolding processes starting from the 4 ns equilibration are shown in Fig. 2. Two major force peaks are observed during the forced-unfolding of CaM N-lobe (Fig. 2A-I) and one major force peak for that of C-lobe (Fig. 2C-I), which are labeled by F_CaN_-max, F_CaN_-c_,_ and F_CaC_-max, respectively. The maximum force peaks F_CaN_-max and F_CaC_-max occurs at extension of ∼75 Å and ∼67 Å respectively, where the two EF-hand motifs of each lobe began to detach from each other, which is confirmed by the breaking of the backbone H-bonds and the decrease of contact area, as shown in Fig. 2A-II,III, 2C-II,III. Representative snapshots of the unfolding process are shown. From the 16.0 ns and the 18.8 ns structural snapshots of Ca^2+^-loaded N-lobe unfolding trajectory (Fig. 2A) and the 15.5 ns and 18.5 ns structural snapshots of Ca^2+^-loaded C-lobe unfolding trajectory (Fig. 2C), it is also found that two EF-hand motifs detach from each other after the appearance of the maximum force peak F_CaN_-max and F_CaC_-max. Therefore we believe that the major force peak, which is thought as the unfolding barrier commonly, comes from the interactions of two EF-hand motifs in both the N- and C-lobes. The force peak F_CaN_-c, which is much less than F_CaN_-max, corresponds to the orientation change of two helices in the EF2 of N-lobe, which could be proved by the 22.4 ns and 24.1 ns structural snapshots in Fig. 2A. It is noticed that two helices in EF1 of N-lobe lost their secondary structure partly, as shown 24.1 ns structural snapshot in Fig. 2A, while these two helices in C-lobe did not, as shown in 18.5 ns structural snapshot in Fig. 2C, which suggests that C-lobe is more stable than N-lobe. Besides, we found that Ca^2+^ did not dissociate from the binding site throughout the unfolding process and the helix-loop-helix motif of EF-hand is mostly kept.

The force-extension curve of the unfolding of isolated Ca^2+^-free N-lobe (Fig. 2B-I) shows two major force peaks, labeled by F_N_-max and F_N_-b, respectively. The force peak F_N_-max coincides with breaking of backbone H-bonds between Ile27 and Ile63, and the force peak F_N_-b comes from the entire loss of contact area between EF1 and EF2 (Fig. 2B-II, III), which can also be observed from the 18.7 ns and the 27.3 ns structural snapshots presented in Fig. 2B. For the isolated Ca^2+^-free C-lobe, there is also one force peak in the force-extension curve which corresponds to the loss of backbone H-bonds between Ile100 and Val136 (Fig. 2D). After this force peak, the contact area of EF3 and EF4 began to decrease slowly. We also noticed that some helices in Ca^2+^-free N-lobe and C-lobe lost their secondary structure in the unfolding processes (30.2 ns snapshot in Fig. 2B and 24.7 ns snapshots in Fig. 2D).

The results for the SMD simulations starting at the 14 ns of the equilibration are shown in Fig. S3. A major force peak is observed for each of the four systems, which indicates an unfolding barrier. They occur at extension of ∼43 Å (Ca^2+^-loaded N lobe), ∼70 Å (Ca^2+^-free N lobe), ∼83 Å (Ca^2+^-loaded C lobe) and ∼97 Å (Ca^2+^-free C lobe), respectively. For Ca^2+^-loaded lobes, the breaking of the backbone H-bonds, the decrease of contact area of two EF-hands, and the major force peak happen synchronously, but not for Ca^2+^-free lobes. These features are similar to the corresponding results starting from the 4 ns of equilibration.

The simulation results indicate that Ca^2+^-binding could affect the unfolding pathway. After the breaking of backbone H-bonds, the contact area decrease to zero quickly for Ca^2+^-loaded systems, but much slower for Ca^2+^-free system. It is also noticed the total extensions of Ca^2+^-free N- and C-lobes systems are larger than that in Ca^2+^-loaded ones at the end of the unfolding simulations due to the firm association of Ca^2+^ ions in Ca^2+^-loaded systems.

### Forced Unfolding of the Full-length CaM

The force-induced unfolding of the full-length Ca^2+^-loaded and Ca^2+^-free CaM were simulated to determine the differences in their unfolding pathway and mimick the corresponding AFM experiments to explain the relevant results [23]. For both Ca^2+^-loaded and Ca^2+^-free CaM, we have performed simulations starting at 4 different snapshots from the equilibration (3.5 ns, 3.75 ns, 4 ns, and 14 ns, respectively). The simulations starting from 3.5 ns, 3.75 ns and 4 ns are performed with pulling velocity 5 Å/ns, and that starting from 14 ns is performed with slower pulling velocity 1 Å/ns.

**Figure 3:**
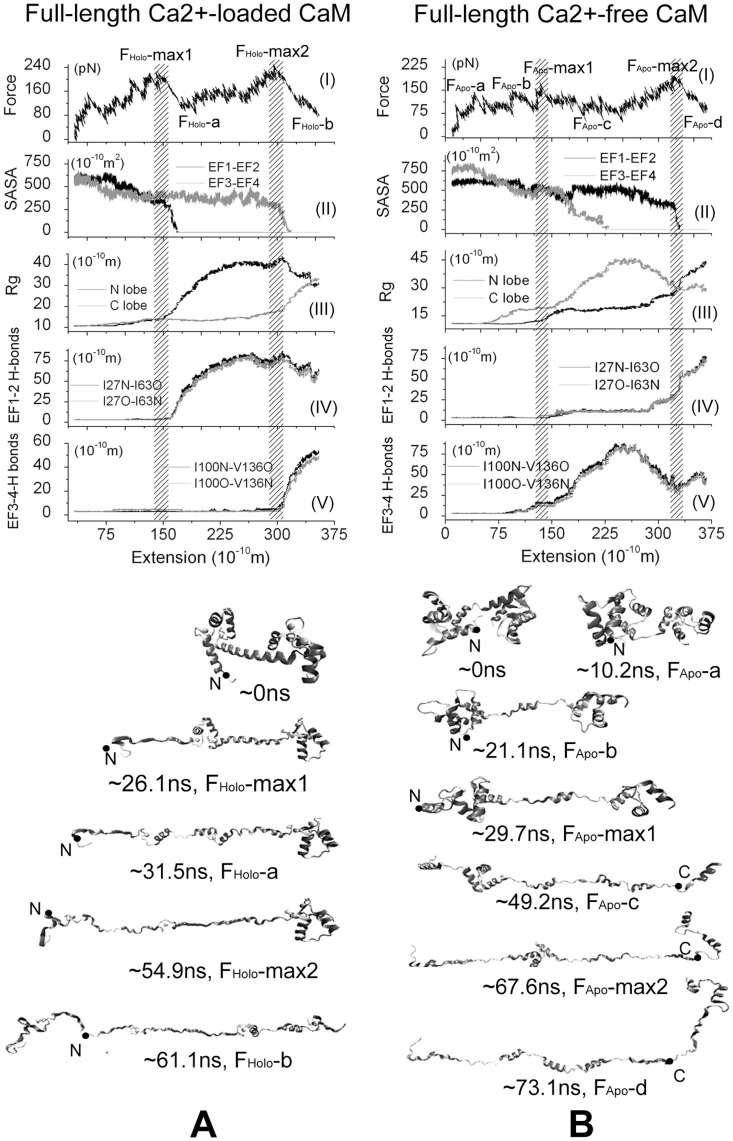
MD simulation results of full-length CaM using 4 ns snapshot of equilibration as the initial structure and ν = 5 Å/ns: A) Ca^2+^-loaded state, and B) Ca^2+^-free state. Above: (I) the force-extension curve; (II) the contact area of two EF-hand motifs in either domain; (III) gyration radius of two domains; and the backbone hydrogen bonds of EFβ-scaffold in N-lobe(IV) and C-lobe(V). Bottom: Five structural snapshots correspond to the special force points labeled in the force-extension curves of Ca^2+^-loaded CaM and seven structural snapshots correspond to the special force points labeled in the force-extension curves of Ca^2+^-free CaM. In all structural snapshots, the N-terminal is at the left side and the C-terminal is at the right side. The forces are applied at the atoms presented with the black solid circle.

The results for the simulation starting from the 4 ns simulations are shown in Fig. 3. There are two main peaks in the force-extension curves of Ca^2+^-loaded CaM that correspond to its two unfolding barriers, which are labeled by F_Holo_-max1 and F_Holo_-max2, respectively. The first main force peak F_Holo_-max1 is located at 150 Å of extension, and the second main force peak F_Holo_-max2 is located at 305 Å of extension (Fig. 3A-I). Monitoring the conformational change of CaM unfolding, it is found that when the first main peak force F_Holo_-max1 occurs, the backbone H-bonds between Ile27 and Ile63 in N-lobe began to vanish (Fig. 3A-IV), and the contact area of EF1 and EF2 droped to zero rapidly (Fig. 3A-II), indicating that the two coupled EF-hand motifs were dissociating. N-lobe unfolded completely after the force peak F_Holo_-max1, as shown in the 26.1 ns and the 31.5 ns structural snapshot in Fig. 3A, which also could be confirmed by the enlargement of its gyration radius (Fig. 3A-III). Meanwhile, C-lobe almost kept its intact structure until the second main force peak F_Holo_-max2 occurred (see the gyration radius change and the 31.5 ns structural snapshot in Fig. 3A). Similar with N-lobe, at the second main force peak F_Holo_-max2, two EF-hand motifs of C-lobe were dissociating, which could be confirmed by breaking of backbone H-bonds between Ile100 and Val136 (Fig. 3A-V), and the loss of contact area of EF3 and EF4 (Fig. 3A-II). After this peak force, C-lobe began to unfold entirely (54.9 ns and the 61.1 ns snapshots in Fig. 3A), which could be also seen from the enlargement of its gyration radius (Fig. 3A-III). Based on these trajectory analyses, it is clear that the two domains unfold in turn where N-lobe unfolds in preference to C-lobe, and both unfolding barriers come from the interaction of two coupled EF-hand motifs for the full-length Ca^2+^-loaded CaM.

For Ca^2+^-free CaM, the unfolding pathway is different with Ca^2+^-loaded CaM and looks more complicated. In addition to the two main force peaks, labeled by F_Apo_-max1 and F_Apo_-max2, there are also some minor force peaks, labeled by F_Apo_-a, F_Apo_-b, as shown in Fig. 3B-I. Observing the trajectory, it is found that at the first force peak F_Apo_-max1, the backbone H-bonds in both N-lobe and C-lobe are broken (Fig. 3B-IV,V), the contact area of EF3 and EF4 began to decrease (Fig. 3B-II), and the gyration radius of C-lobe began to increase largely (Fig. 3B-III), which suggested that the coupled EF3 and EF4 was dissociating and C-lobe was unfolding (29.7 ns and 49.2 ns structural snapshots in Fig. 3B). Through the backbone H-bonds in N-lobe has been broken, but their length did not increase continually and the contact area of EF1 and EF2 was hold till the second major force peak F_Apo_-max2 occurred, which indicated the N-lobe did not unfold entirely after the first force peak. After F_Apo_-max2, the N-lobe began to unfold completely, which is also indicated by the entire loss of the contact area of EF1 and EF2 (Fig. 3B-II), the enlargement of gyration radius of N-lobe (Fig. 3B-III), the 67.6 ns and the 73.1 ns structural snapshots in Fig.3B. Therefore, we concluded that the major force peaks also resulted in overcoming the interactions of EF-hand motifs for Ca^2+^-free CaM, similar as that happened in Ca^2+^-loaded CaM case. Different with the unfolding pathway of Ca^2+^-loaded CaM, the first major force peak corresponds to the unfolding of C-lobe and the second comes from the unfolding of N-lobe. Besides, it is observed that F_Apo_-a corresponds to the reorientation of N-lobe (10.2 ns snapshots in Fig. 3B) and F_Apo_-b corresponds to the unfolding of first helix in C-lobe (21.1 ns snapshots in Fig. 3B). It is also noticed that breaking of backbone H-bonds and the loss of the contact area between two EF-hand motifs in either domain of CaM almost happened at the same time in Ca^2+^-loaded CaM, but happened asynchronously in Ca^2+^-free CaM, which was similar as the case in isolated CaM domains.

The SMD results starting from 3.5 ns and 3.75 ns of equilibration show the similar features as that from 4 ns of equilibration for both Ca^2+^-loaded and Ca^2+^-free CaM. For the simulations of Ca^2+^-loaded CaM (Fig. S4A for results starting from 3.5 ns and Fig. S5A for results starting from 3.75 ns), two unfolding energy barriers corresponding to the unfolding of N- and C-lobes are also found, located at the ∼125 and ∼260 Å for the simulations starting from 3.5 ns, and at ∼130 and ∼245 Å for the simulations starting from 3.75 ns. That peak forces are synchronous with the break of backbone H-bond of EFβ-scaffold and the enlargement of gyration radius of each lobe. For Ca^2+^-free CaM, two major and two minor force peaks are found in both simulations (Fig. S4B for results starting from 3.5 ns and Fig. S5B for results starting from 3.75 ns). The two major force peaks, located at ∼105 and ∼280 Å (Fig. S4B), and at ∼90 and ∼290 Å (Fig. S5B) respectively, indicate two unfolding energy barriers. After conquering these two barriers, the C-lobe and N-lobe began to unfold successively. The two minor force peaks result from the unfolding of helix and/or reorientation of domain.

To accomplish unfolding within time scale available in MD simulations, pulling velocity much faster than that used in real single-molecule experiments are utilized. To test whether the fast pulling velocity may lead to artifact in the unfolding pathway, we performed simulations with slower velocity (1 Å/ns) starting from the 14 ns snapshots of the full-length Ca^2+^-loaded and Ca^2+^-free CaM equilibration, the results are shown in Fig. S6. For both cases, the unfolding pathways are very similar with that of pulling velocity 5 Å/ns described above. There are two major force peaks for both systems, which happen at the extension of ∼143 Å and ∼275 Å for Ca^2+^-loaded CaM, and at ∼118 and ∼297 for Ca^2+^-free CaM. The two lobes of CaM unfold sequentially and the unfolding of each lobe results in a major force peak. Moreover, the major force peaks is related to both the backbone H-bonds and the contact area of two EF-hands for Ca^2+^-loaded CaM, but not for Ca^2+^-free CaM. Because of the low pulling velocity, the fluctuation of the applied forces become stronger and the peak value of the forces reduced.

### Effect of the Pulling Directions

**Figure 4:**
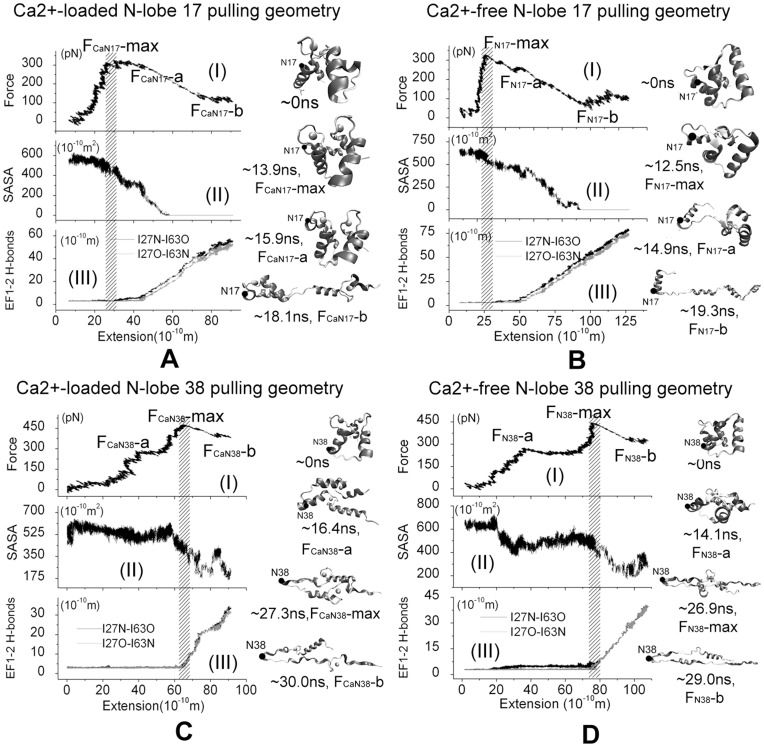
MD simulation results of force-induced unfolding of isolated Ca^2+^-loaded and Ca^2+^-free N-lobe unfolding with non-N-C-terminal pulling directions using 4 ns snapshot of equilibration as the initial structure and ν = 5 Å/ns. External forces were applied on the N-lobe residue 17 (A, B) or residue 38(C, D) to unfold the sequence between the constrained C-terminal and the residue where force is applied. Left: (I) Force-extension curve; (II) Contact area between EF1 and EF2 of N-lobe; and (III) Backbone hydrogen bonds of EFβ-scaffold coupled EF1 and EF2. Right: snapshots of the unfolding trajectories. The external force was applied at the atoms presented with black solid circle (residues 17 or 38), and the C-terminal residue 74 is constrained.

The unfolding processes of the isolated N-lobe were investigated further by applying force in two different pulling geometries to study the effect of different pulling geometries to the unfolding pathway and explain the recent AFM experiments [28]. The force profiles and snapshots of the force-induced unfolding process are showed in Fig.4.

For the N-lobe 17 pulling scheme, the Cα atom of N-lobe C-terminal residue is constrained and force was applied on the Cα atom of residue 17 instead of the N-terminal. In Ca^2+^-loaded cases, there is one major force peak, labeled by F_CaN17_-max, at the extension of ∼35 Å in the force-extension curves and this force peak looks more flat than those in N-C-terminal pulling scheme (Fig. 4A-III). At the time force reaches F_CaN17_-max, one hydrogen bond between the EF-hand motifs is broken and there is a decrease for EF1-EF2 contact area. At the force point of F_CaN17_-a, another hydrogen bond is broken and the contact area between EF1 and EF2 hands begin to vanish. Therefore it is believed that this flat force peak is used to conquer the interaction of two EF-hand motifs, which could be also seen from the ∼13.9 ns, 15.2 ns and ∼18.0 ns snapshots in Fig. 4A. Because residues 1–17 can not be extended in this case, the full extension of the protein is obviously smaller than that in the N-C-terminal pulling scheme, as shown the ∼18.0 ns snapshots in Fig. 4A. For Ca^2+^-free N-lobe, again one major peak force, labeled by F_N17_-max, were found at the extension of 30 Å in the force-extension curve (Fig. 4B-III), when the two helixes of EF1 motif dissociate (12.5 ns, 14.9 ns snapshot, Fig. 4B) and one hydrogen bond is broken (Fig. 4B-I). At the force point of F_CaN17_-a, another backbone H-bond is broken and the contact area of EF1 and EF2 decreases obviously. Until the force point F_N17_-b, two EF-hand motifs do not lost their contact completely (19.3 ns snapshot, Fig. 4B).

For the N-lobe 38 pulling scheme, the Cα atom of N-lobe C-terminal residue was constrained and force was applied on the Cα atom of residue 38. In this case, a major force peak, labeled by F_CaN38_-max or F_N38_-max, could also be found in each force-extension curve of either Ca^2+^-loaded or Ca^2+^-free N-lobe (Fig. 4C-III, 4D-III). At both force peaks, the backbone H-bonds are broken and the contact area of EF1 and EF2 decreases obviously, as shown in Fig.4C-I,II) and Fig.4D-I,II). The force peak also results from conquering the interaction of two EF-hand motifs. Different with other cases, the contact of two EF-hand motifs did not lost completely when the system is extended almost fully (Fig.4C-II and Fig.4D-II). This is because residue 38 is located on the flexible loop region between the two EF-hand motifs and the external force can not affect the first EF-hand motif directly. Observing the unfolding trajectories, it is found that two EF-hand motifs of N-lobe is almost in parallel to each other till the extension reach its maximum (see 30.0 ns and 29.0 ns snapshots for Ca^2+^-loaded and Ca^2+^-free N-lobe respectively). After both major peaks, the external forces did not decrease largely because the peptide between the pulled atom and constrained atom becomes almost linear except the helix structures which require larger forces to unfold.

## Discussions and Conclusions

The force-induced Ca^2+^-loaded CaM unfolding in its isolated and full-length state has been investigated experimentally with the single molecule AFM techniques [23], [28] It is shown that there is a major energy barrier in the force-induced unfolding of each isolated CaM domain and there exist two distinct force peaks in the extension-force curves of the full-length CaM which correspond to the unfolding of two globular CaM domains which can be characterized by the difference extension length fitted with the wormlike chain model. Our SMD simulations successfully reproduced the unfolding process of Ca^2+^-loaded CaM and revealed the detailed pathways in Ca^2+^-loaded CaM unfolding processes. Some fine-tuned force peaks, which are responsible for the helix unfolding and reorientation of the globular domain, also are found in the simulation results which were not visible in AFM studies due to the spatial and force resolution of the experiments. The maximum forces to unfold the CaM constructs in our simulations are about 10 times larger than those observed in the AFM experiments. This results in the much larger loading rates used in our simulations to enable the completion of the unfolding in the nano-second time scale than that used in the experiments (almost 10^8^ times larger in our simulations). Similar phenomena have also been observed in the experimental and simulated force-induced unfolding of other protein domains such as titin [25], [38]. Our simulations revealed the detailed conformational changes of Ca^2+^-loaded CaM unfolding at the atomistic-level, and it is concluded that the two peak forces observed in both AFM experiments and our simulations should result in the conquering of the interactions between the two EF-hands motif in either domain, including the backbone hydrogen bond of EFβ-scaffold and the direct contact of the two motifs.

The force-extension curves of Ca^2+^-loaded CaM unfolding obtained by our simulation not only agree with Rief’s study in 2009, but is similar with Ikai’s study in 2002. The latter presented that the unfolding pathway of Ca^2+^-loaded and Ca^2+^-free CaM are different and Ca^2+^-binding could change the stability of CaM, but not analyzed the unfolding pathway of CaM. The force-extension curves of Ca^2+^-free CaM obtained by our SMD simulation, which has two main peak forces, are not fit Ikai’s results completely, in which there is only one big peak force in the force curve. We thought that it is compatible because the pulling speed in SMD simulation is almost 10^8^ times larger than that in AFM experiment and the large pulling speed could not provide the time enough to relax the CaM protein during unfolding process, therefore produced some other peak force. Furthermore, the first main force peak is much smaller than the second one in the simulation results. Therefore it is possible to be ignored by the AFM experiment because of its small value and the slow pulling speed.

Comparing the N-C-terminal and the non-N-C-terminal pulling geometries, it is found that the maximum unfolding force of the former is obviously larger, in good agreement with the AFM experimental results [28]. The maximum force occurred at different extension for the two kinds of non-N-C-terminal pulling geometries, which is an straightforward outcome related with the relative location of the pulled and the constrained residues/atoms When the Cα atom of residue 17 was pulled, because the residue is nearby the first Ca^2+^-binding loop which starts from residue 21, the pulling force will not interrupt or have very little effect on the interaction between the two helixes of EF1 motif, therefore these two helix did not dissociate completely and the extension of system is limited compared with the N-C-terminal pulling scheme. Similarly because residue 38 located at the connected region of the two EF-band motifs, when its Cα atom was pulled, the main interaction between two EF-hand motifs did break, though their contact did not lost completely because both two EF-hand motifs align with force and parallel with each other, thus the final extension of N-lobe is only half of its full extension. Our results confirmed that locations of pulled and constrained atoms, which defined the pulling direction, could affect the unfolding pathway partially, although generally the pulling direction could not change the main steps to overcoming the unfolding barrier, which includes the interactions of two EF-hand motif in the case of CaM lobes.

Our simulations revealed that the two lobes of CaM unfold sequentially and Ca^2+^-binding could change the unfolding order of two CaM lobes. The C-lobe is more stable in Ca^2+^-binding CaM, whereareas the N-lobe is more stable than without the Ca^2+^-binding, the results are in agreement with the experimental chemical (urea) and thermal denaturation data [16], [17]. That is, although Ca^2+^ binding might affect the stability of both domains, the effect on C domain is more intense and stronger than that on N domain. Moreover, the unfolding pathway and total extension are also different for Ca^2+^-loaded and Ca^2+^-free system. If Ca^2+^-loaded, in both the isolated domains and the full-length CaM, the breaking of hydrogen bond in EFβ-scaffold, the decreasing of the SASA between two EF-hand motifs, and the force peak happened at almost the same time. But this is not the case for the Ca^2+^-free system. In the Ca^2+^-loaded condition, five residues in EF-hand loop coordinate with Ca^2+^, which maintains the structural integrity in the vicinity and prevents the metal ion coordination motif to unfold completely.

The simulation results suggest that the backbone H bonds and the hydrophobic interactions in Ile27-Ile63 and Ile100-Val136 are critical for the force-induced CaM unfolding and these interactions result in the unfolding barrier (Fig. 3 and Fig. S7). Actually, these residues are reserved in whole CaM family. Some studies have been done to investigate the function of Ile27, Ile63, Ile100 and Val136. The single-residue mutation experiments of these four residues showed that the conformation of CaM and the thermal stability could be altered dramatically. They also suggest that the integrity of both the apo- and holo-forms of Cam is important for the maintenance of its biological function and confirm the importance of conserving the structural function of the residues involved in the *β*-sheet interactions [35]. The Ca^2+^ titration of ^15^N-labeled mutant V136G CaM presented that the mutated C-domain is a mixture of unfolded, partially folded (site III occupied), and native-like folded (sites III and IV occupied) conformations [39]. What’s more, NMR measurements of selectively labeled [^15^N]Ile CaM also proved that thermal stability of the folding units is affected by the mutation experiment of Ile 27, 63, 100 [40]. Combining the experimental results mentioned above and our simulation results, we believed that the reason that these residues are related to the thermal stability of CaM is that the interactions between these residues are responsible to the unfolding barrier of CaM. Besides these two pair of residues, we also found that there are other interactions between hydrophilic residues which are responsible to the unfolding barrier, which is the interaction of Leu32, Met36 and Leu48, Met51, Ile52 in N lobe, and of Leu105, Met109 and Val121, Met124, Leu125 in C-lobe, as shown in Fig. S7. So we suggest that these residues also are important for the thermal stability of CaM. Further mutation experiments need to be done to validate their influence on the thermal stability of CaM.

The function of CaM is implemented by binding Ca^2+^ which could induce the conformational change of CaM and expose its hydrophobic patch to interact with the CaM-binding proteins. As we discussed above, we suggested that Ca^2+^ not only promote the conformational change of CaM, but change its thermal stability. We guess that the thermal stability change of CaM by Ca^2+^-binding also play a part in the CaM binding to its target protein, because it is inconceivable to image that CaM binds to its target appropriately using a relative loose and flexible secondary structure.

In conclusion, the force-induced unfolding of CaM is simulated using both the isolated domains and the full-length CaM. Our results reveal that two domains of CaM would unfold sequentially and Ca^2+^-binding could change the stability of two domains, consequently reverse their unfolding order. Our atomistic-level results are in good agreement with of the experimental results performed on Ca^2+^-loaded CaM with AFM and give reasonable interpretation of force-extension curves of Ca^2+^-loaded CaM, that is, the main unfolding barrier comes from the interaction of two EF-motifs. The location and direction of pulling may results in partially unfolded states and also may change the unfolding pathways.

## Supporting Information

Figure S1 Two non-N-C-terminal pulling schemes for the isolated Ca^2+^-loaded and Ca^2+^-free N-lobe, in which the Cα atom of C-terminal residues 74 is constrained, and the Cα atom of residues 17 and 38 was set to the point of pulling force application. The cyan balls represent the Cα atoms, and the green balls represent the Ca^2+^ ions.
(TIF)

Figure S2 Backbone RMSD of six systems-ApoN(black), ApoC(red), HoloN(green), HoloC (blue), Apo(cyan), Holo(purple) from their initial structures during the equilibration period.
(TIF)

Figure S3 MD simulation results of the isolated CaM domains using 14 ns snapshot of equilibration as the initial structure and ν = 5 Å/ns : A) Ca^2+^-loaded N-lobe, B) Ca^2+^-free N-lobe, C) Ca^2+^-loaded C-lobe, D) Ca^2+^-free C-lobe. On the left, (I): Force-extension curve; (II) Contact area of two EF-hand motifs; (III) Backbone hydrogen bonds of EFβ-scaffold coupled two EF-hand motifs. On the snapshots of the forced-unfolding processes, N-terminal is presented with the cyan ball and the Ca^2+^ ions are presented by the green ball.
(TIF)

Figure S4 SMD simulation results of full-length CaM using the 3.5 ns snapshot of equilibration as the initial structure and ν = 5 Å/ns: A) Ca^2+^-loaded state, and B) Ca^2+^-free state. From top to bottom, the panels are the force-extension curve; the contact area of two EF-hand motifs in either domain; gyration radius of two domains; and the backbone hydrogen bonds of EFβ-scaffold in N-lobe and C-lobe, respectively. Five structural snapshots correspond to the special force points labeled in the force-extension curves of Ca^2+^-loaded CaM and Ca^2+^-free CaM, respectively. In all structural snapshots, the N-terminal is presented with the cyan ball and the Ca^2+^ ions are presented by the green ball.
(TIF)

Figure S5 SMD simulation results of full-length CaM using the 3.75 ns snapshot of equilibration as the initial structure and ν = 5 Å/ns: A) Ca^2+^-loaded state, and B) Ca^2+^-free state. From top to bottom, the panels are the force-extension curve; the contact area of two EF-hand motifs in either domain; gyration radius of two domains; and the backbone hydrogen bonds of EFβ-scaffold in N-lobe and C-lobe, respectively. Five structural snapshots correspond to the special force points labeled in the force-extension curves of Ca^2+^-loaded CaM and Ca^2+^-free CaM, respectively. In all structural snapshots, the N-terminal is presented with the cyan ball and the Ca^2+^ ions are presented by the green ball.
(TIF)

Figure S6 SMD simulation results of full-length CaM using the 14 ns snapshot of equilibration as the initial structure and ν = 1 Å/ns: A) Ca^2+^-loaded state, and B) Ca^2+^-free state. From top to bottom, the panels are the force-extension curve; the contact area of two EF-hand motifs in either domain; gyration radius of two domains; and the backbone hydrogen bonds of EFβ-scaffold in N-lobe and C-lobe, respectively. Several structural snapshots correspond to the special force points labeled in the force-extension curves of Ca^2+^-loaded CaM and Ca^2+^-free CaM, respectively. In all structural snapshots, the N-terminal is presented with the cyan ball and the Ca^2+^ ions are presented by the green ball.
(TIF)

Figure S7 Contact area change of some key residues during the CaM unfolding simulation using the last snapshots of equilibration period as the initial structure: A) Ca^2+^-loaded state, and B) Ca^2+^-free state.
(TIF)

Table S1 The detailed information of the six systems used in our simulation.
(DOC)

Table S2 Hydrogen bond* in EFβ-scaffold during the equilibration period
(DOC)

Table S3 The contact areas (SASA) of two EF-hand motifs in a domain, and the gyration radius of EF-hand motif during the equilibration period
(DOC)
