# Supplementary figures and images for: The Ca2+ Influence on Calmodulin Unfolding Pathway: A Steered Molecular Dynamics Simulation Study
Source: PLoS One. 2012 Nov 7;7(11):e49013. doi: 10.1371/journal.pone.0049013 (PMC3492193; doi:10.1371/journal.pone.0049013)

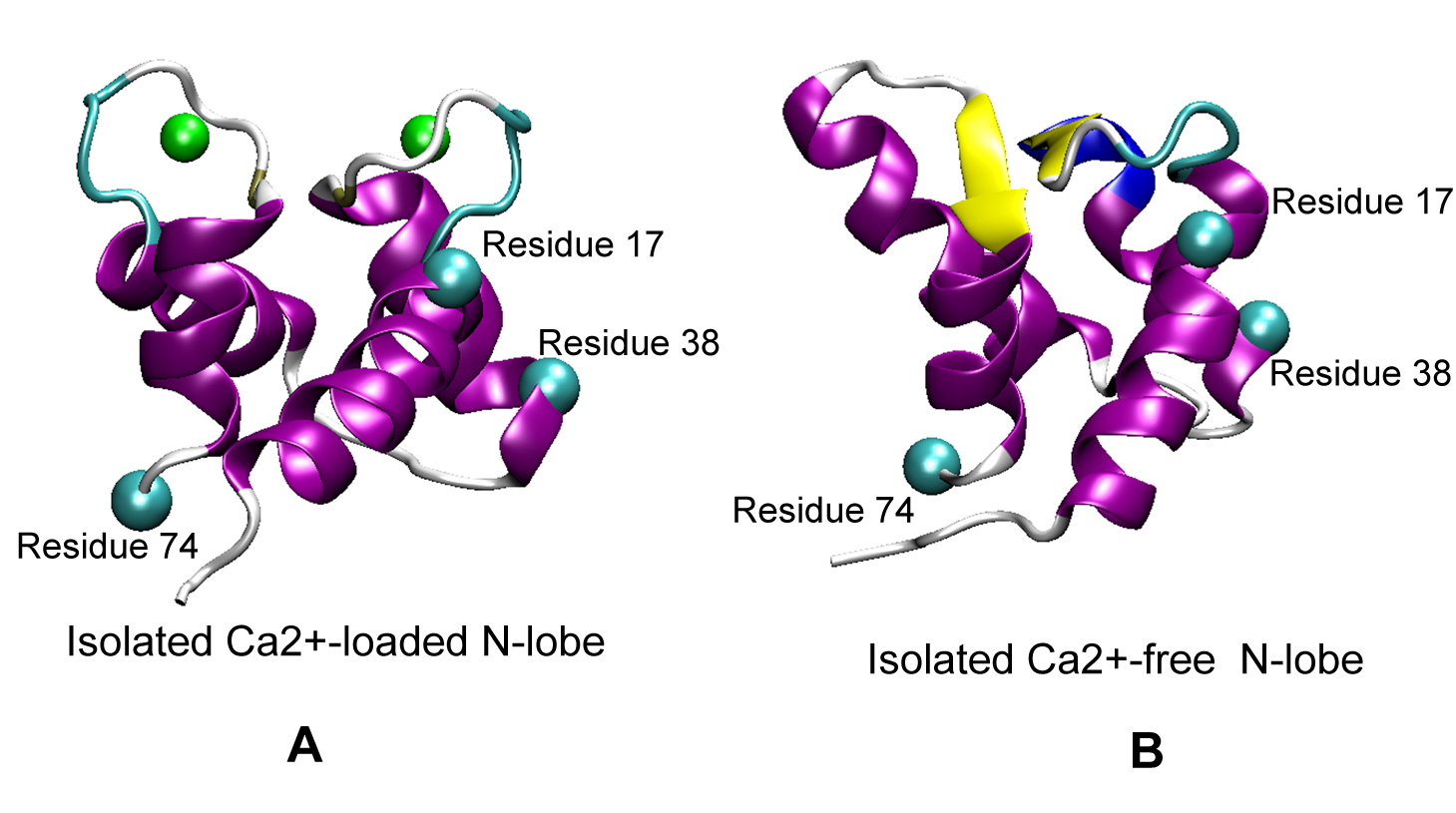

Supplement: Figure S1 — Two non-N-C-terminal pulling schemes for the isolated Ca2+-loaded and Ca2+-free N-lobe, in which the Cα atom of C-terminal residues 74 is constrained, and the Cα atom of residues 17 and 38 was set to the point of pulling force application. The cyan balls represent the Cα atoms, and the green balls represent the Ca2+ ions. (TIF) [file pone.0049013.s001.tif]

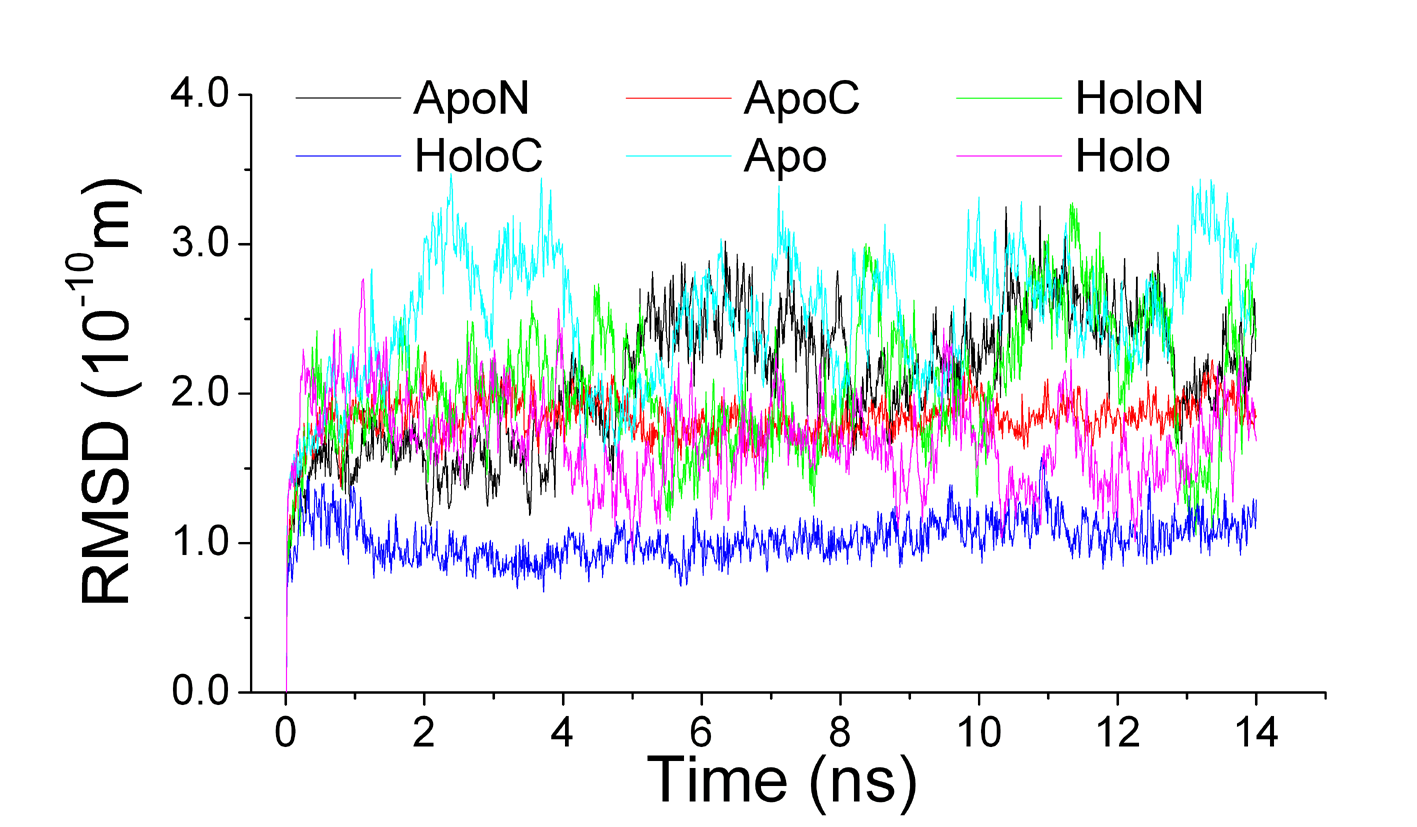

Supplement: Figure S2 — Backbone RMSD of six systems-ApoN(black), ApoC(red), HoloN(green), HoloC (blue), Apo(cyan), Holo(purple) from their initial structures during the equilibration period. (TIF) [file pone.0049013.s002.tif]

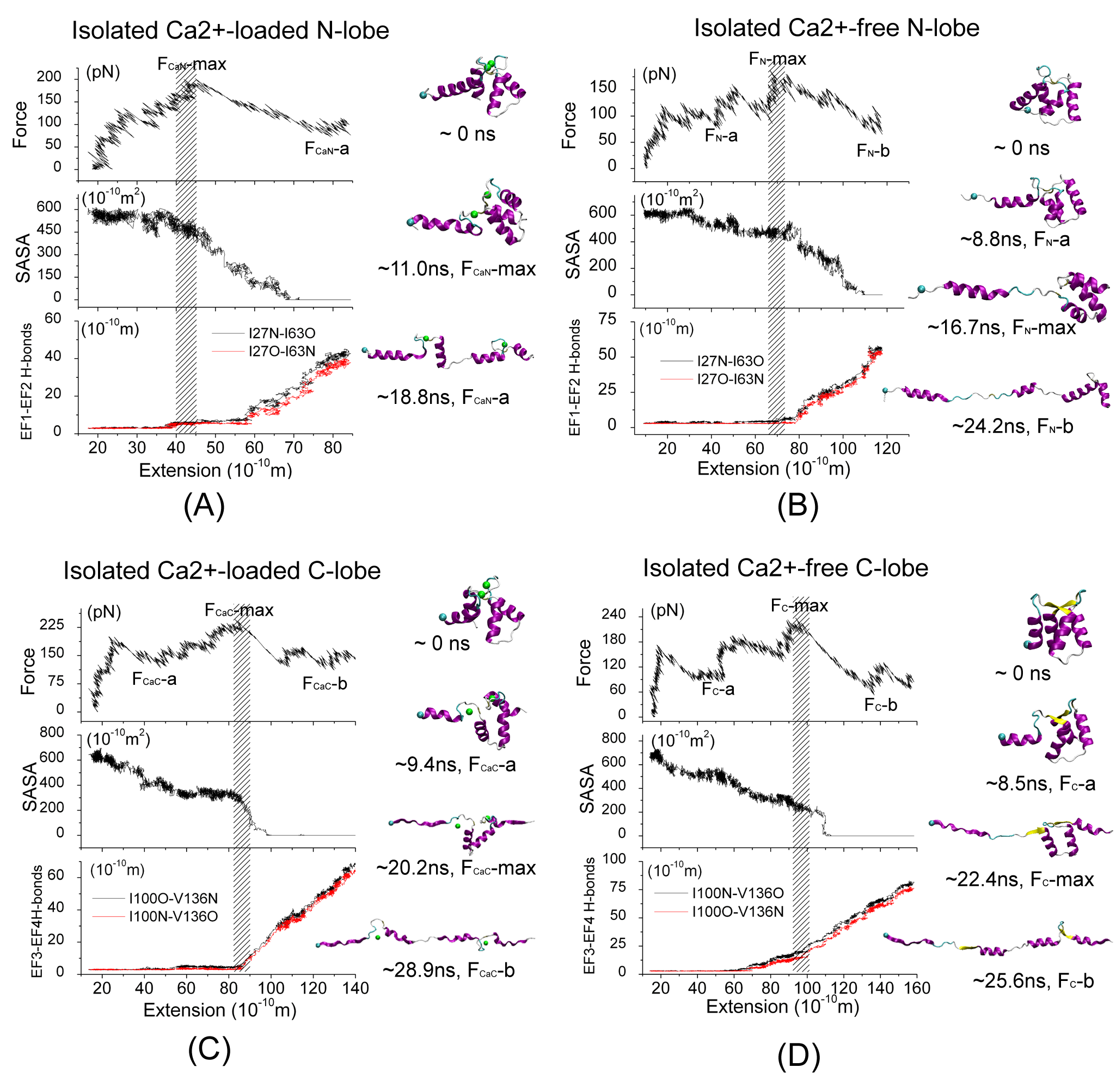

Supplement: Figure S3 — MD simulation results of the isolated CaM domains using 14 ns snapshot of equilibration as the initial structure and ν = 5 Å/ns : A) Ca2+-loaded N-lobe, B) Ca2+-free N-lobe, C) Ca2+-loaded C-lobe, D) Ca2+-free C-lobe. On the left, (I): Force-extension curve; (II) Contact area of two EF-hand motifs; (III) Backbone hydrogen bonds of EFβ-scaffold coupled two EF-hand motifs. On the snapshots of the forced-unfolding processes, N-terminal is presented with the cyan ball and the Ca2+ ions are presented by the green ball. (TIF) [file pone.0049013.s003.tif]

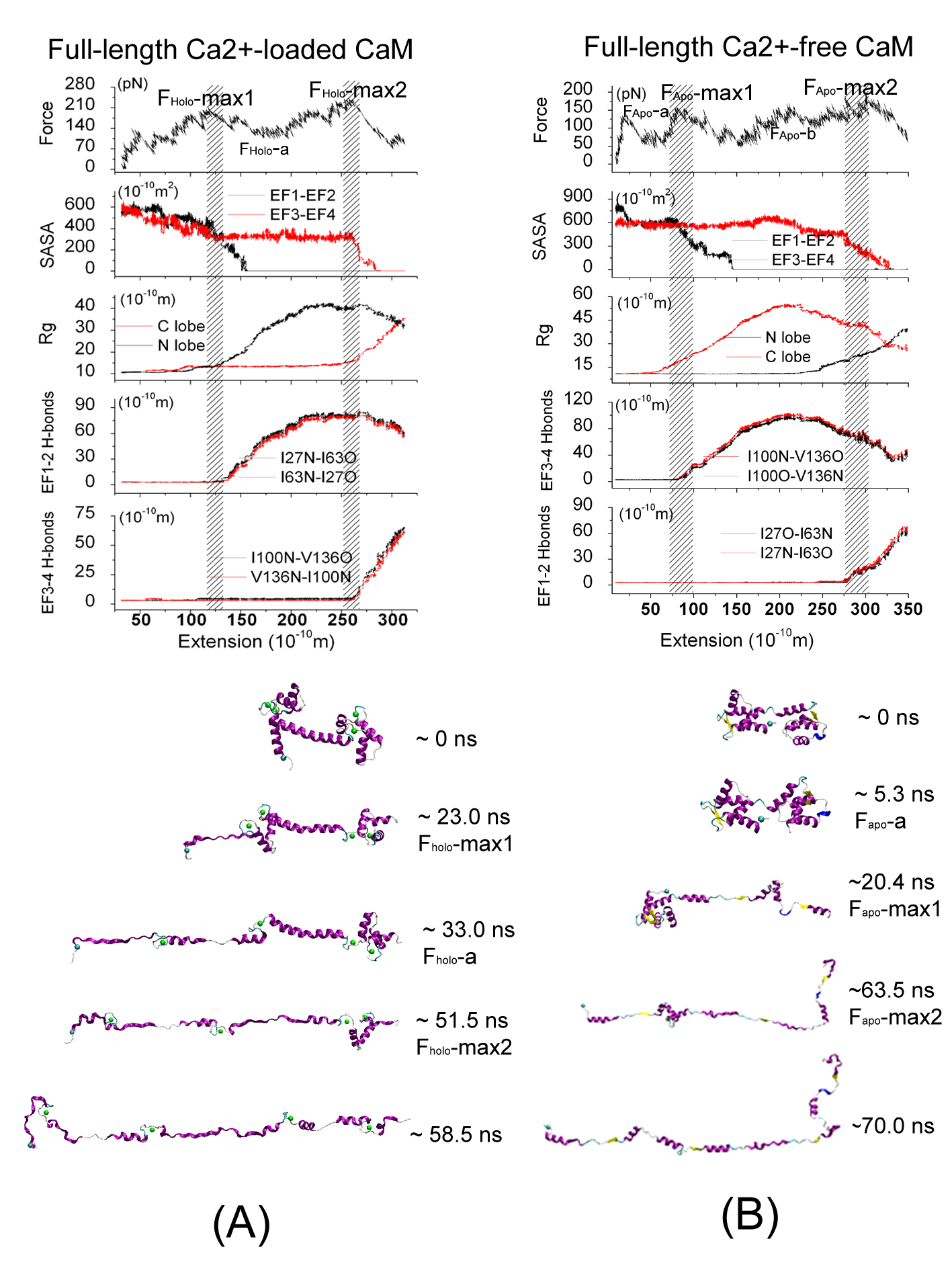

Supplement: Figure S4 — SMD simulation results of full-length CaM using the 3.5 ns snapshot of equilibration as the initial structure and ν = 5 Å/ns: A) Ca2+-loaded state, and B) Ca2+-free state. From top to bottom, the panels are the force-extension curve; the contact area of two EF-hand motifs in either domain; gyration radius of two domains; and the backbone hydrogen bonds of EFβ-scaffold in N-lobe and C-lobe, respectively. Five structural snapshots correspond to the special force points labeled in the force-extension curves of Ca2+-loaded CaM and Ca2+-free CaM, respectively. In all structural snapshots, the N-terminal is presented with the cyan ball and the Ca2+ ions are presented by the green ball. (TIF) [file pone.0049013.s004.tif]

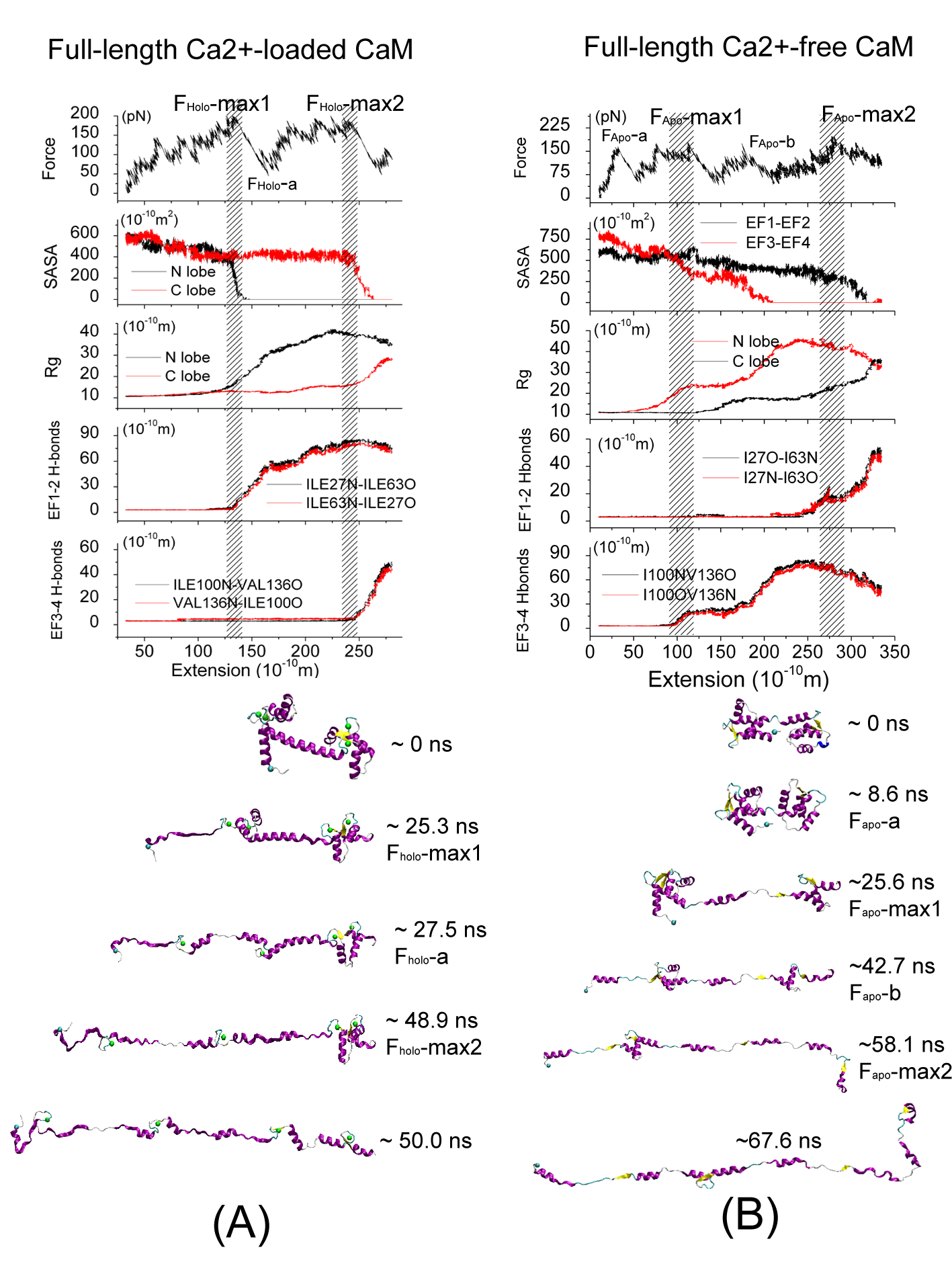

Supplement: Figure S5 — SMD simulation results of full-length CaM using the 3.75 ns snapshot of equilibration as the initial structure and ν = 5 Å/ns: A) Ca2+-loaded state, and B) Ca2+-free state. From top to bottom, the panels are the force-extension curve; the contact area of two EF-hand motifs in either domain; gyration radius of two domains; and the backbone hydrogen bonds of EFβ-scaffold in N-lobe and C-lobe, respectively. Five structural snapshots correspond to the special force points labeled in the force-extension curves of Ca2+-loaded CaM and Ca2+-free CaM, respectively. In all structural snapshots, the N-terminal is presented with the cyan ball and the Ca2+ ions are presented by the green ball. (TIF) [file pone.0049013.s005.tif]

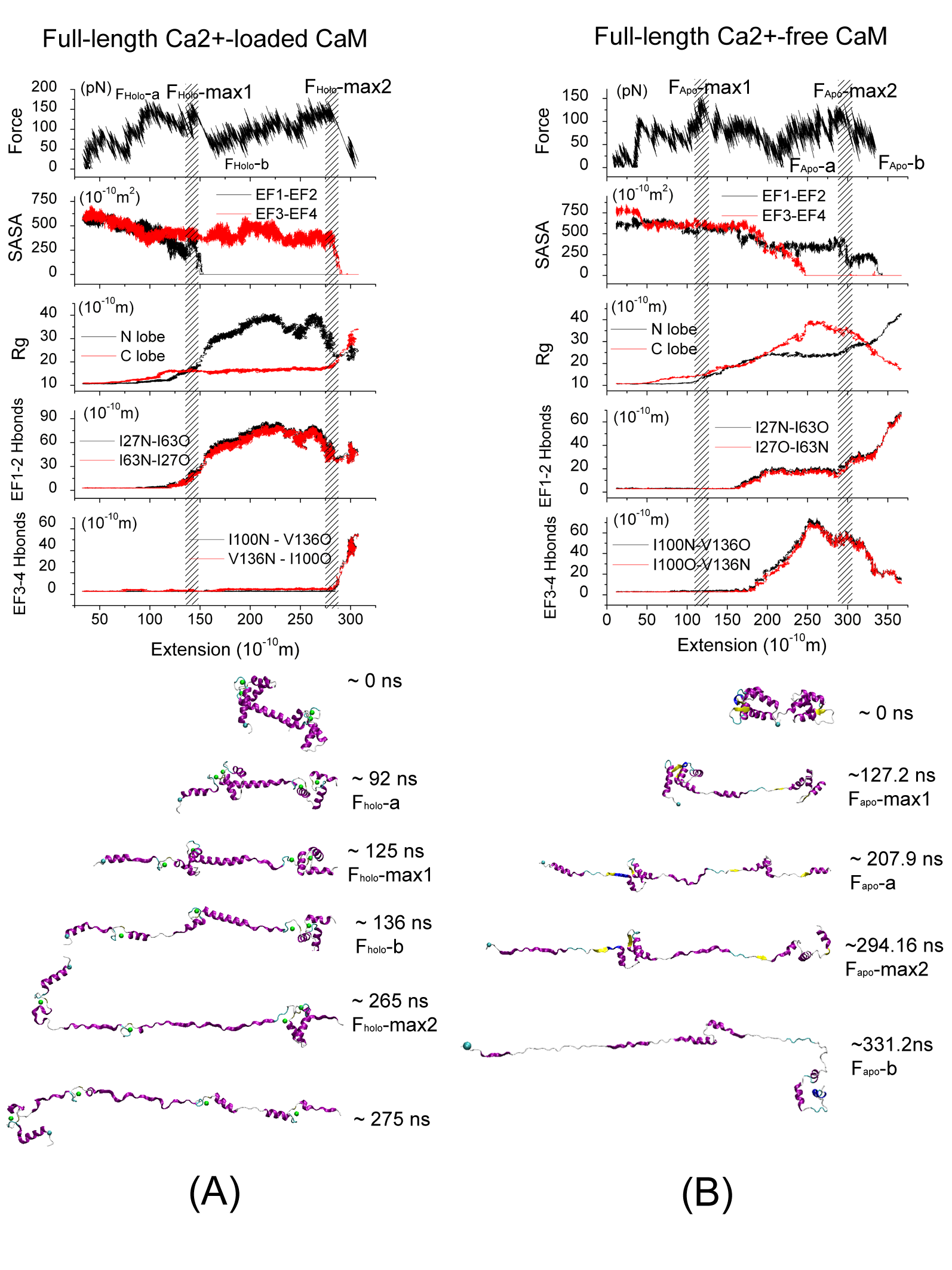

Supplement: Figure S6 — SMD simulation results of full-length CaM using the 14 ns snapshot of equilibration as the initial structure and ν = 1 Å/ns: A) Ca2+-loaded state, and B) Ca2+-free state. From top to bottom, the panels are the force-extension curve; the contact area of two EF-hand motifs in either domain; gyration radius of two domains; and the backbone hydrogen bonds of EFβ-scaffold in N-lobe and C-lobe, respectively. Several structural snapshots correspond to the special force points labeled in the force-extension curves of Ca2+-loaded CaM and Ca2+-free CaM, respectively. In all structural snapshots, the N-terminal is presented with the cyan ball and the Ca2+ ions are presented by the green ball. (TIF) [file pone.0049013.s006.tif]

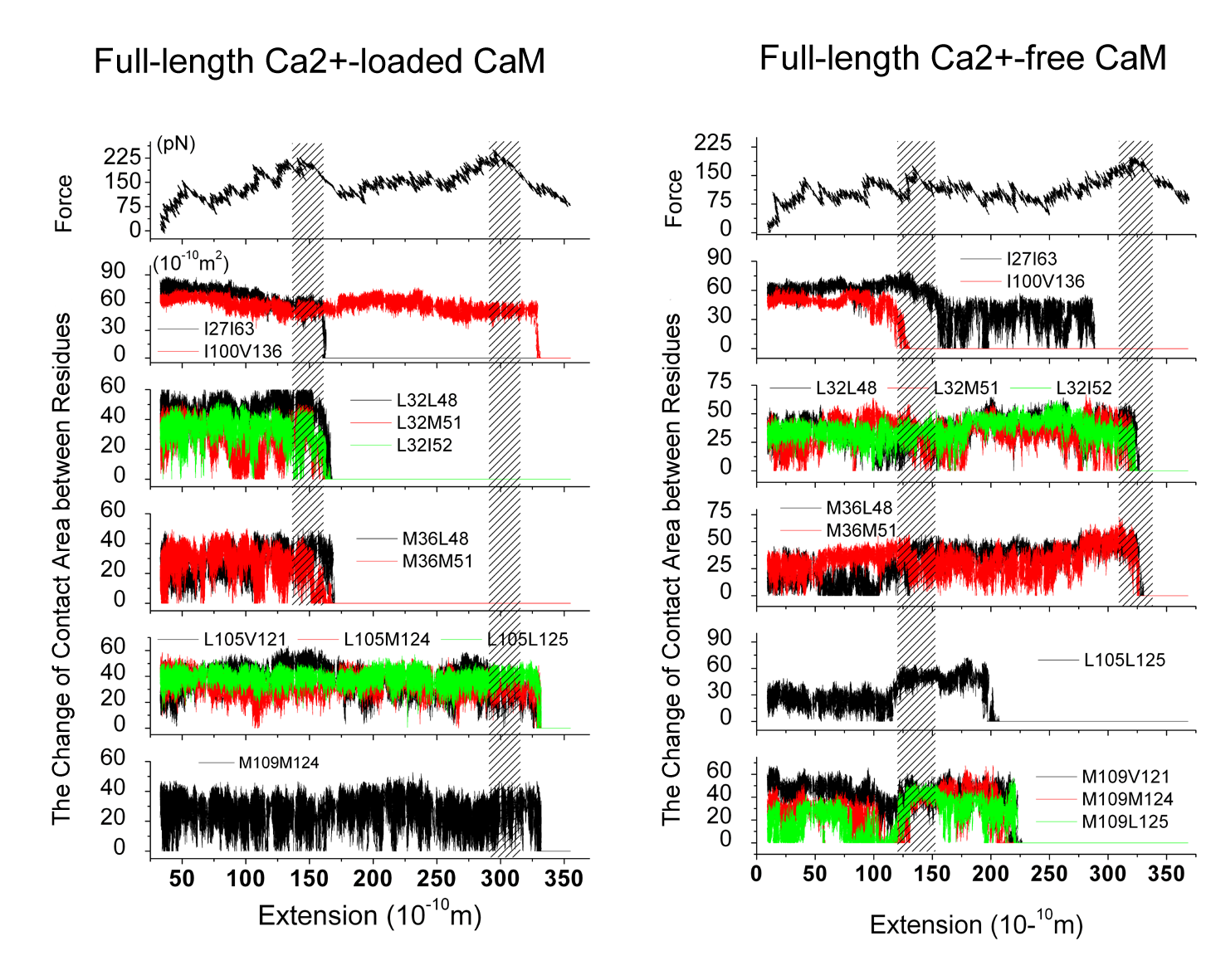

Supplement: Figure S7 — Contact area change of some key residues during the CaM unfolding simulation using the last snapshots of equilibration period as the initial structure: A) Ca2+-loaded state, and B) Ca2+-free state. (TIF) [file pone.0049013.s007.tif]
