# Supplementary material for: The Ca2+ Influence on Calmodulin Unfolding Pathway: A Steered Molecular Dynamics Simulation Study
Source: PLoS One. 2012 Nov 7;7(11):e49013. doi: 10.1371/journal.pone.0049013 (PMC3492193; doi:10.1371/journal.pone.0049013)
Supplement: Table S1 — The detailed information of the six systems used in our simulation. (DOC) [file pone.0049013.s008.doc]

**Table S1, Th**e detailed component of six systems

|  | | Holo Calmodulin | | | Apo Calmodulin | | |
| --- | --- | --- | --- | --- | --- | --- | --- |
|  | | Isolated N-lobe | Isolated C-lobe | Full-length | Isolated N-lobe | Isolated C-lobe | Full-length |
| Number of Atoms | Protein | 1114 | 1030 | 2266 | 1112 | 1028 | 2262 |
|  | counterions | 54 | 43 | 110 | 54 | 39 | 72 |
|  | Water | 58305 | 45759 | 117336 | 56943 | 42081 | 76197 |
|  | All | 59473 | 46832 | 119712 | 58109 | 43148 | 78531 |
| Box dimension(Å) | | 202*56*55 | 170*50*57 | 271*75*61 | 198*60*52 | 168*56*49 | 252*52*63 |
