# Supplementary material for: The Ca2+ Influence on Calmodulin Unfolding Pathway: A Steered Molecular Dynamics Simulation Study
Source: PLoS One. 2012 Nov 7;7(11):e49013. doi: 10.1371/journal.pone.0049013 (PMC3492193; doi:10.1371/journal.pone.0049013)
Supplement: Table S2 — Hydrogen bond* in EFβ-scaffold during the equilibration period (DOC) [file pone.0049013.s009.doc]

**Table S2, Hydrogen bond* in EFβ-scaffold during the equilibration period**

|  | | Calculated value | Experimental value^#^ |
| --- | --- | --- | --- |
| Apo CaM | Isolated N-lobe | I27-N : I63-O (2.96±0.16 Å)  T29-N : G61-O (3.35±0.47 Å)  I63-N : I27-O (2.88±0.16 Å) | I27-N : I63-O (2.83Å)  T29-N : G61-O (3.33Å)  I63-N : I27-O (2.80Å)  I100-N : V136-O (3.44Å)  V136-N : I100-O (3.22Å) |
|  | Isolated C-lobe | I100-N : V136-O (2.99±0.17 Å)  V136-N : I100-O (2.92±0.18 Å)  Y138-N : G98-O (3.21±0.47 Å) |  |
|  | Full-length | I27-N : I63-O (3.02±0.18 Å)  T29-N : G61-O (3.07±0.24Å)  I63-N : I27-O (2.86±0.14Å)  I100-N : V136-O (3.07±0.16Å)  V136-N : I100-O (2.81±0.12Å) |  |
| Holo CaM | Isolated N-lobe | I27-N : I63-O (2.90±0.15 Å)  I63-N : I27-O (3.05±0.26 Å) | I27-N : I63-O (2.63Å)  I63-N : I27-O (2.94Å)  I100-N : V136-O (2.84Å)  V136-N : I100-O (2.96Å) |
|  | Isolated C-lobe | I100-N : V136-O (2.95±0.15 Å)  V136-N : I100-O (3.10±0.10 Å) |  |
|  | Full-length | I27-N : I63-O (2.85±0.19Å)  I63-N : I27-O (3.01±0.20Å)  I100-N : V136-O (2.89±0.14Å)  V136-N : I100-O (2.97±0.16Å) |  |

* Hydrogen bond: X…H-X <3.5Å, X…H-X >150°

# The values of holo conformation is from X-ray structure of Ca^2+^/CaM (PDB code : 1CLL (9) ), The values of apo conformation is from NMR structure of CaM (PDB code :1CFD (10) )
