# Supplementary material for: The Ca2+ Influence on Calmodulin Unfolding Pathway: A Steered Molecular Dynamics Simulation Study
Source: PLoS One. 2012 Nov 7;7(11):e49013. doi: 10.1371/journal.pone.0049013 (PMC3492193; doi:10.1371/journal.pone.0049013)
Supplement: Table S3 — The contact areas (SASA) of two EF-hand motifs in a domain, and the gyration radius of EF-hand motif during the equilibration period (DOC) [file pone.0049013.s010.doc]

**Table S3, The contact areas (SASA) of two EF-hand motifs in a domain, and the gyration radius of EF-hand motif during the equilibration period**

|  | | SASA(Å^2^) | | gyration radius(Å) | |
| --- | --- | --- | --- | --- | --- |
|  | | Calculated | Experimental* | Calculated | Experimental* |
| Apo CaM | Isolated N-lobe | 699.24±23.95 | EF1-EF2: 677.66  EF3-EF4: 684.31 | 10.46±0.10 (EF1) 9.37±0.20(EF2) | 11.35 (EF1) 10.35 (EF2)  8.95 (EF3) 9.70 (EF4) |
|  | Isolated C-lobe | 734.16±30.31 |  | 9.23±0.11 (EF3) 9.59±0.09 (EF4) |  |
|  | Full-length | EF1-EF2: 670.08±20.11  EF3-EF4: 685.83±24.57 |  | 10.56±0.10 (EF1) 9.52±0.15 (EF2)  9.28±0.07 (EF3) 9.91±0.11 (EF4) |  |
| Holo CaM | Isolated N-lobe | 580.15±25.74 | EF1-EF2: 612.06  EF3-EF4: 619.50 | 11.76±0.15 (EF1) 9.07±0.16 (EF2) | 11.88 (EF1) 9.43 (EF2)  9.77 (EF3) 9.83 (EF4) |
|  | Isolated C-lobe | 639.84±22.78 |  | 9.72±0.11 (EF3) 9.98±0.12 (EF4) |  |
|  | Full-length | EF1-EF2: 610.47±29.95  EF3-EF4: 602.45±26.24 |  | 11.88±0.14 (EF1) 9.14±0.21 (EF2)  9.65±0.10 (EF3) 9.98±0.12 (EF4) |  |

* The experimental values of holo conformation is calculated from X-ray structure of Ca^2+^/CaM (PDB code : 1CLL (9)), The experimental values of apo conformation is calculated from NMR structure of CaM (PDB code :1CFD (10))
